# Supplementary material for: Imputation of label-free quantitative mass spectrometry-based proteomics data using self-supervised deep learning
Source: Nat Commun. 2024 Jun 26;15:5405. doi: 10.1038/s41467-024-48711-5 (PMC11208500; doi:10.1038/s41467-024-48711-5)
Supplement: Supplementary file 1 — Supplementary Information [file 41467_2024_48711_MOESM1_ESM.pdf]

Supplementary Material for "Imputation of label-free quantitative mass spectrometry-based proteomics data using self-supervised deep learning"

Authors

Henry Webel[1,2], Lili Niu[1], Annelaura Bach Nielsen[1], Marie Locard-Paulet[1,3], Matthias Mann[1,4], Lars Juhl Jensen[1], Simon Rasmussen[1,2,5,*]

Affiliations

[1] Novo Nordisk Foundation Center for Protein Research, Faculty of Health and Medical Sciences, University of Copenhagen, Copenhagen N, Denmark
[2] Novo Nordisk Foundation Center for Basic Metabolic Research, Faculty of Health and Medical Sciences, University of Copenhagen, Copenhagen N, Denmark
[3] Institut de Pharmacologie et de Biologie Structurale (IPBS), Université de Toulouse, CNRS, Université Toulouse III - Paul Sabatier (UT3), Toulouse, France
[4] Department of Proteomics and Signal Transduction, Max Planck Institute of Biochemistry, Martinsried, Germany
[5] The Novo Nordisk Foundation Center for Genomic Mechanisms of Disease, Broad Institute of MIT and Harvard, Cambridge, MA 02142, USA

Keywords

Imputation, self-supervised learning, deep learning, reproducibility, mass spectrometry, proteomics

Supplementary Figures

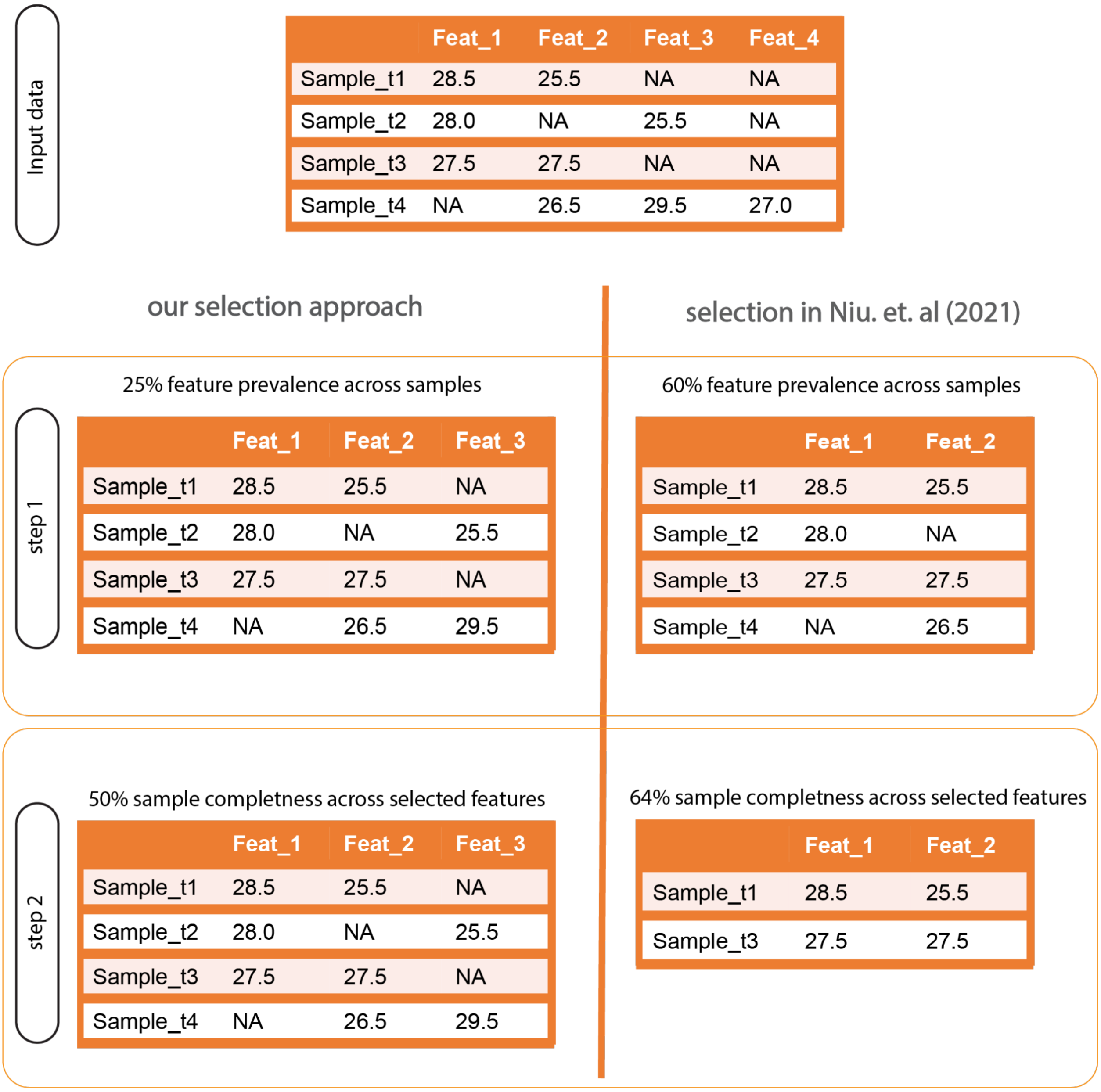

Supplementary Figure 1. Comparison between approaches used here and random shifted normal (RSN) imputation for ALD data. First, features are independently selected by their completeness over a set of samples. Second, samples are selected over the set of selected features from the first step. As we were aiming to impute as many features as possible, we lowered the threshold below 60% in the first step. However, we needed enough observations from a single feature to be able to learn the dependence on other features which worked with a threshold of one quarter in larger data sets (which can be adapted). The second step ensures that samples have some coverage over the prevalent features, i.e. low quality samples get removed, as imputation is done based on other features in a sample.

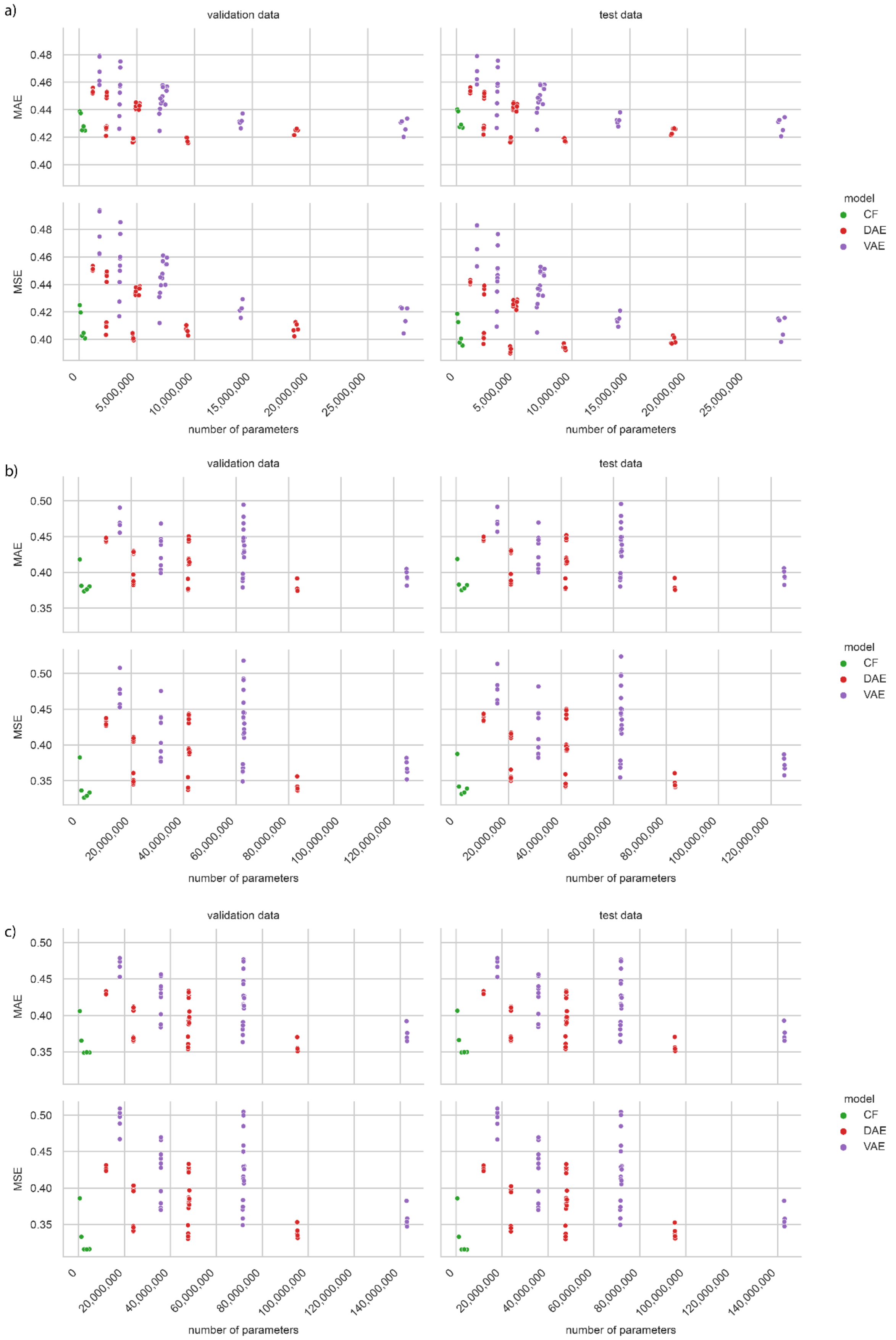

Supplementary Figure 2. Grid search performances by number of model parameters. The total number of parameters depends on the architectures (which are the same, see Methods) and the number of features. CF performs more stable than DAE and VAE which show more variation in their performance for **a)** protein groups **b)** aggregated peptides **c)** precursors. Mean average error (MAE) and mean squared error (MSE) of simulated missing values for both validation and test data.

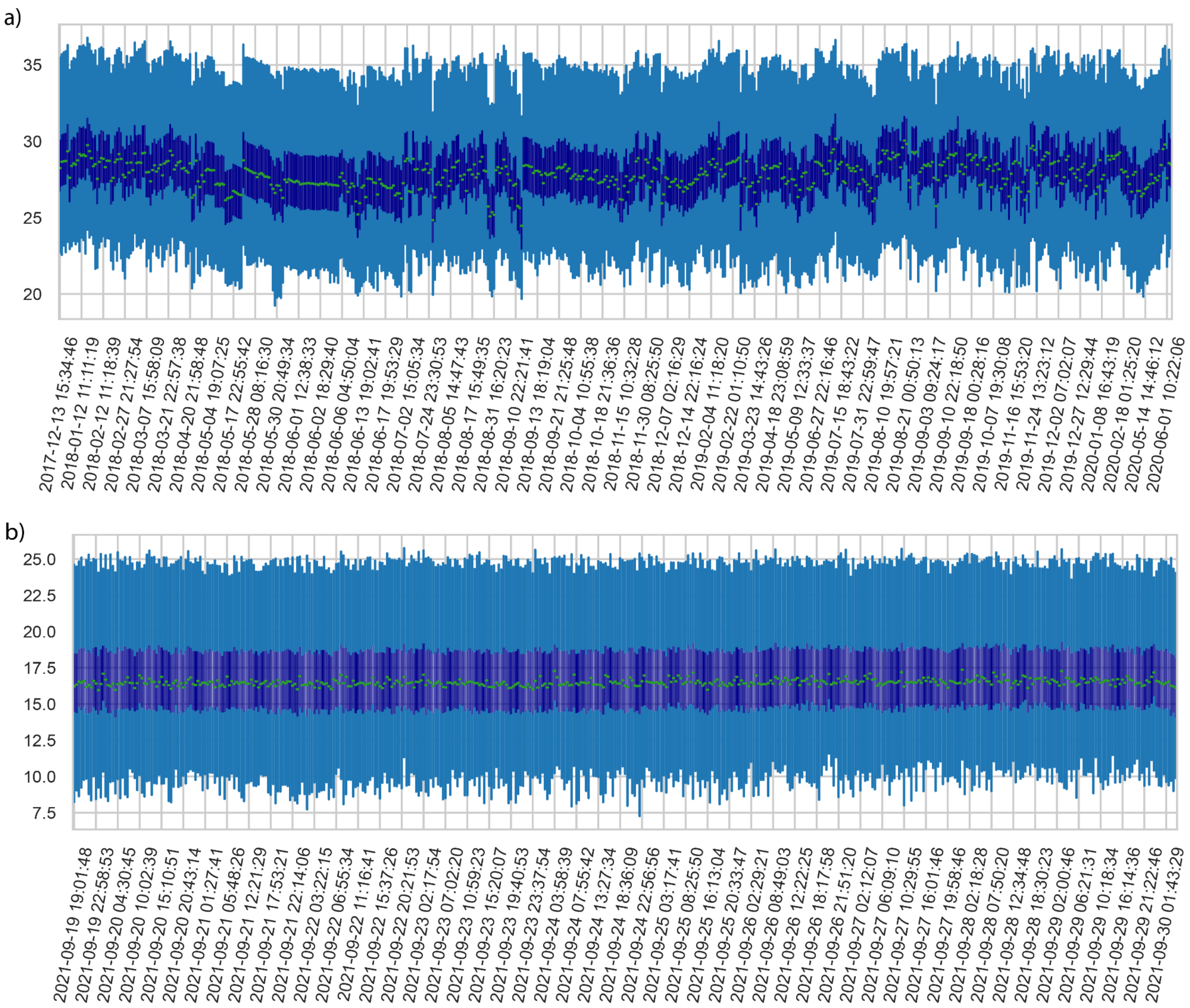

Supplementary Figure 3. Boxplots of each of the HeLa samples for instrument 6070 and ALD plasma sample dataset a) Boxplot of logarithm two transformed intensities for protein groups (y-axis) of HeLa data for instrument 6070. Notice the long time range of samples and that intensities range roughly from 20 to 36. **b)** Boxplot of logarithm two transformed intensities (y-axis) of plasma samples measured in comparison to a) short time range of several weeks. The values range from 8 to 26. The lower and upper hinges (dark blue) of the boxplots correspond to the first and third quartiles. The upper and lower whiskers (light blue) extend from the hinge to the highest and lowest values, respectively, but no further than 1.5 times the interquartile range from the hinge. Data beyond the ends of whiskers are outliers and are plotted individually.

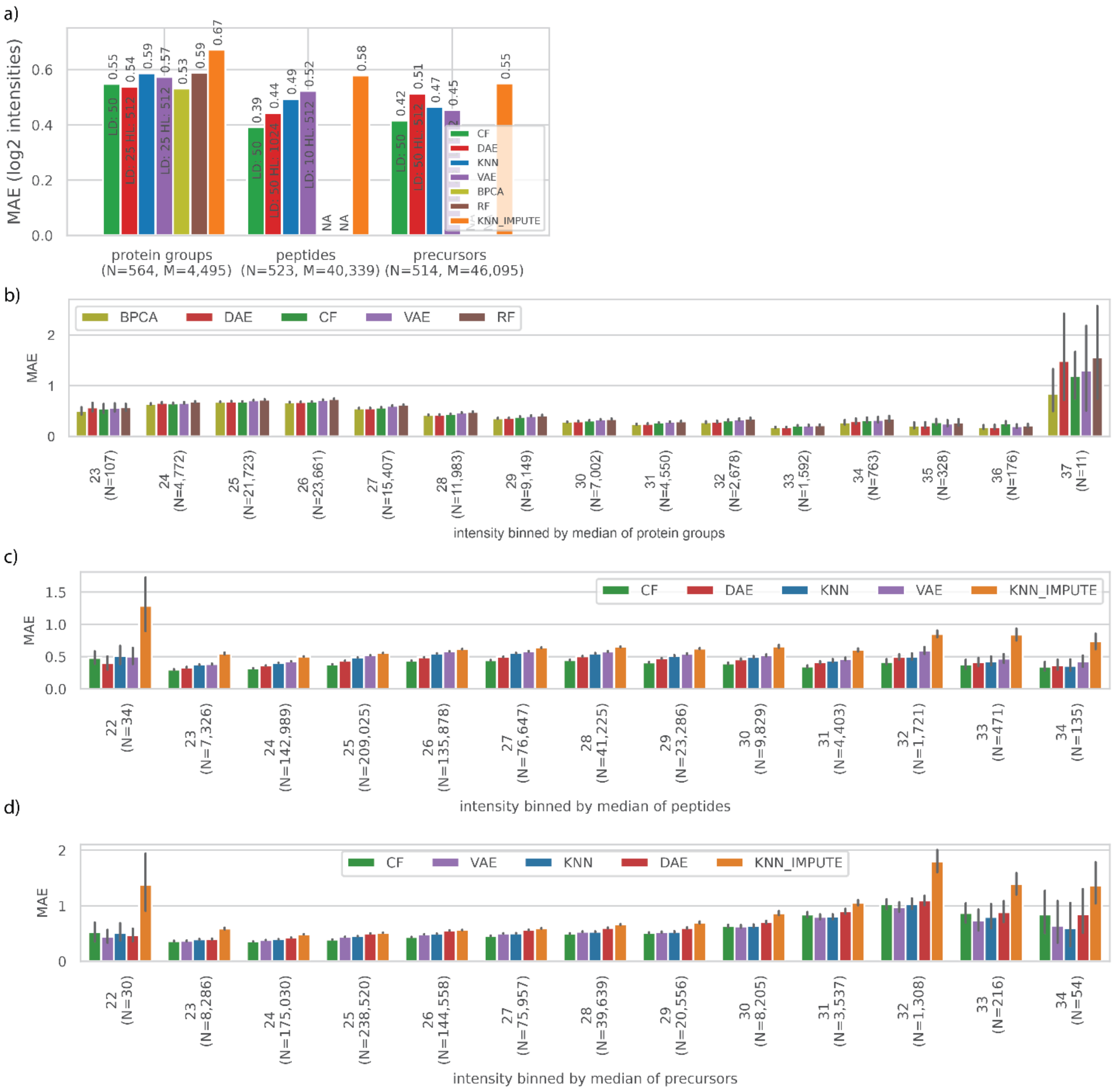

Supplementary Figure 4. Performance for larger dataset for best models according to grid search. a) Performance of imputation methods at the level of protein groups, aggregated peptides, and precursors. Mean averaged error is shown on the y-axis (green: CF, red: DAE, blue: KNN (scikit-learn), purple: VAE, olive: BPCA, brown: random forest (missForest), orange: KNN_IMPUTE (impute)). The ML methods performed competitively in comparison to the other models. **b)** MAE 95% bootstrapped confidence interval for protein groups intensities in test split binned by the integer value of their median intensity in the training data split (4,495 protein groups, based on 103,902 intensities, models ordered by overall performance, 1000 draws to compute the confidence interval) **c)** as b) for peptides (40,399 peptides, based on 652,968 intensities, models ordered by overall performance, 1000 draws to compute the confidence interval) **d)** as b) for precursors (46,095 precursors, based on 715,896 intensities, models ordered by overall performance, 1000 draws to compute the confidence interval).

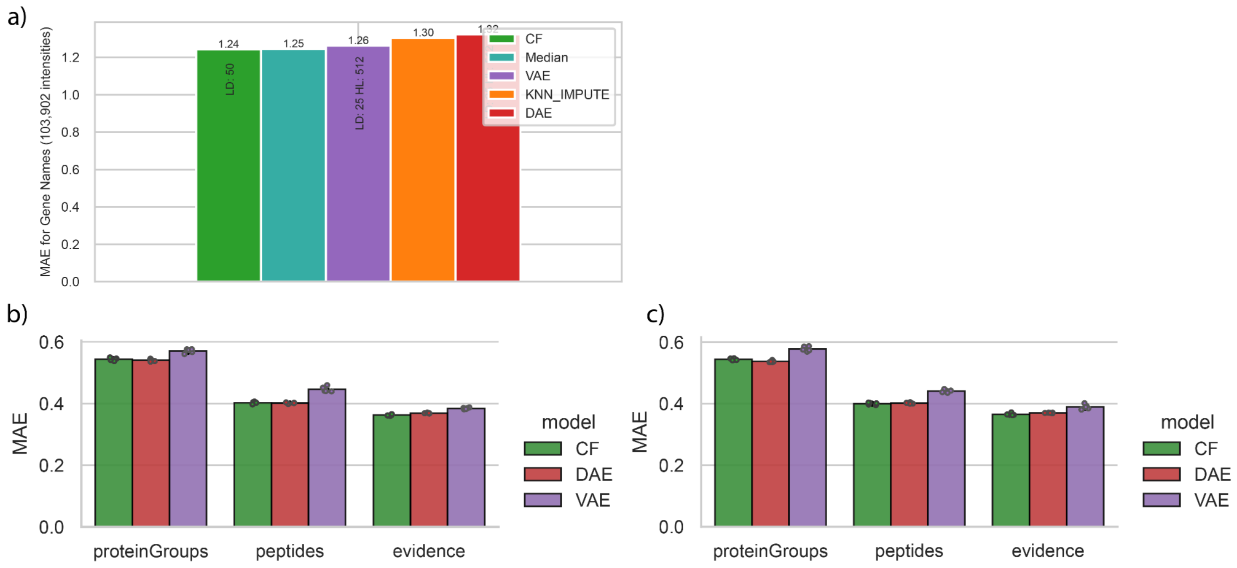

Supplementary Figure 5. Overfitting analysis on large development dataset a) Fitting large development dataset to randomly permuted protein groups. Median performance is not changed due to permutation and will be close to the best performance on the permuted data. **b)** Repeated training of the best self-supervised deep learning models on different data splits and **c)** repeat training only on the same random split of the data. In both cases the overall performance is stable, indicated by the bootstrapped 95% confidence interval using 1000 draws. All analysis was done with a share of 25 percent MNAR in simulated missing values. See **Supplementary Data 5** for summary statistics.

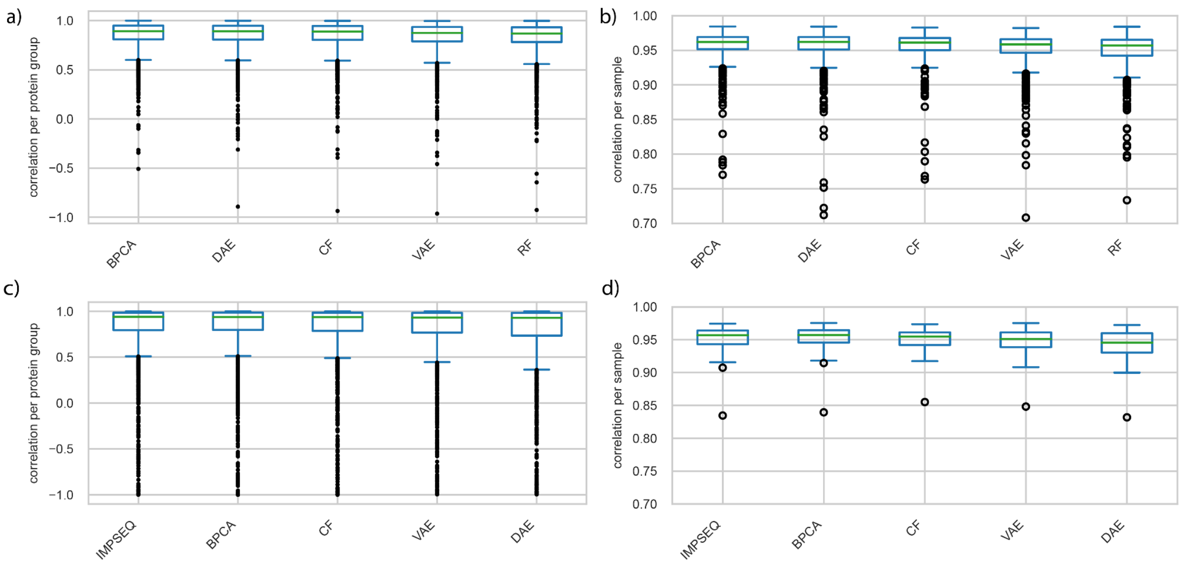

Supplementary Figure 6. Boxplots of correlations between protein groups. a) Correlation between 4,494 protein groups with at least 3 intensities in test data split with 25 percent MNAR for large development dataset and c) 1,566 protein groups for small development dataset. b) Correlations between protein groups between 564 samples and d) 50 samples. Mean correlation lies around 0.96 for all methods. The box of the boxplots extends from the first quartile to the third quartile of the correlations with their median as a separate line. The whiskers extend no more than one and a half times the interquartile range, ending at the farthest correlation within that interval. Outliers are plotted as separate dots. See **Supplementary Data 6** for summary statistics.

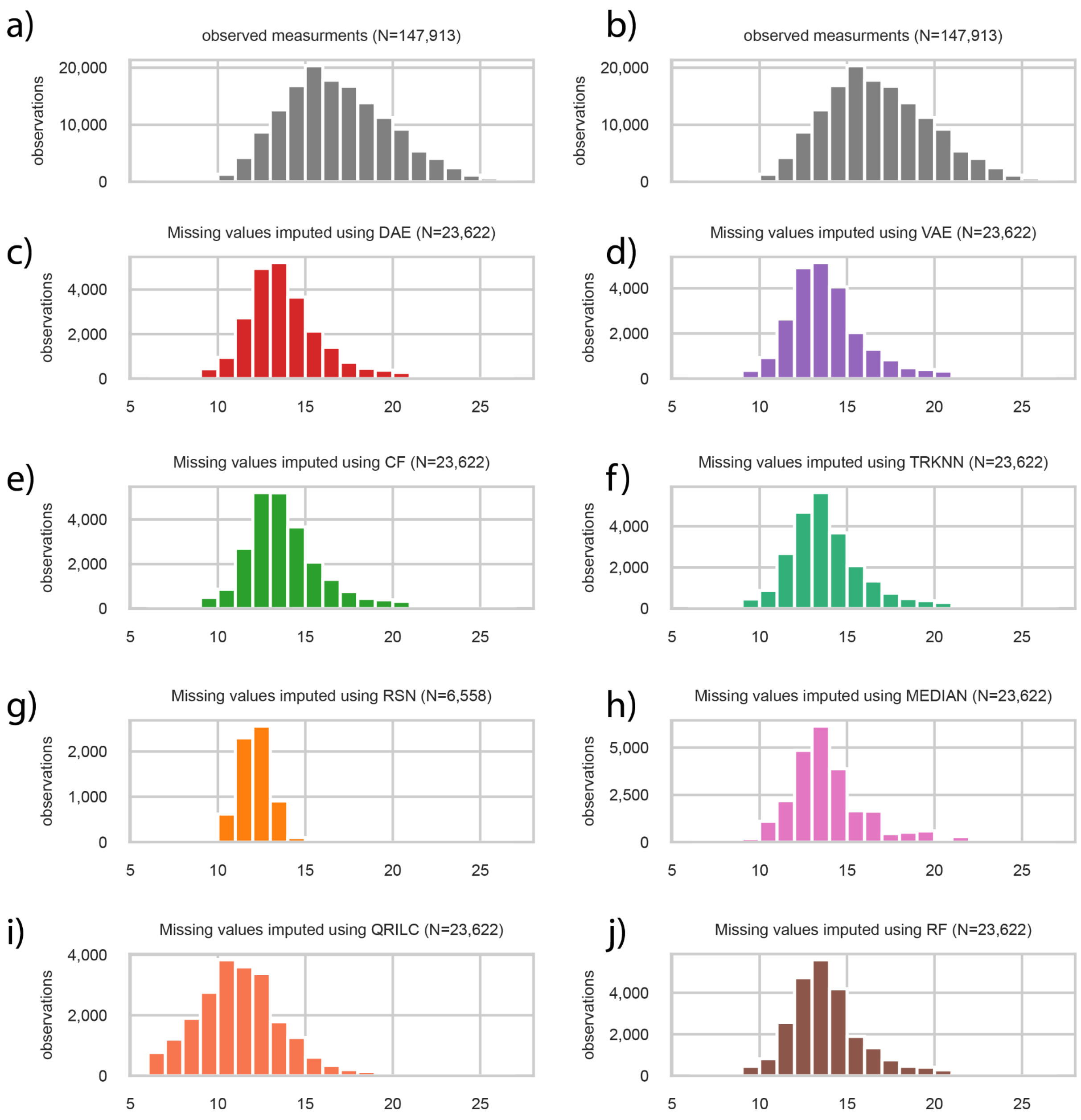

Supplementary Figure 7. ALD plasma protein groups imputations. a-b) Histogram of all quantified protein group measurements which were available in gray, displayed twice in the first row. **c-j)** Histograms of all model based imputations. **g)** RSN was only used to impute a subset of the features as in the original study. All histogram bars overlap exactly.

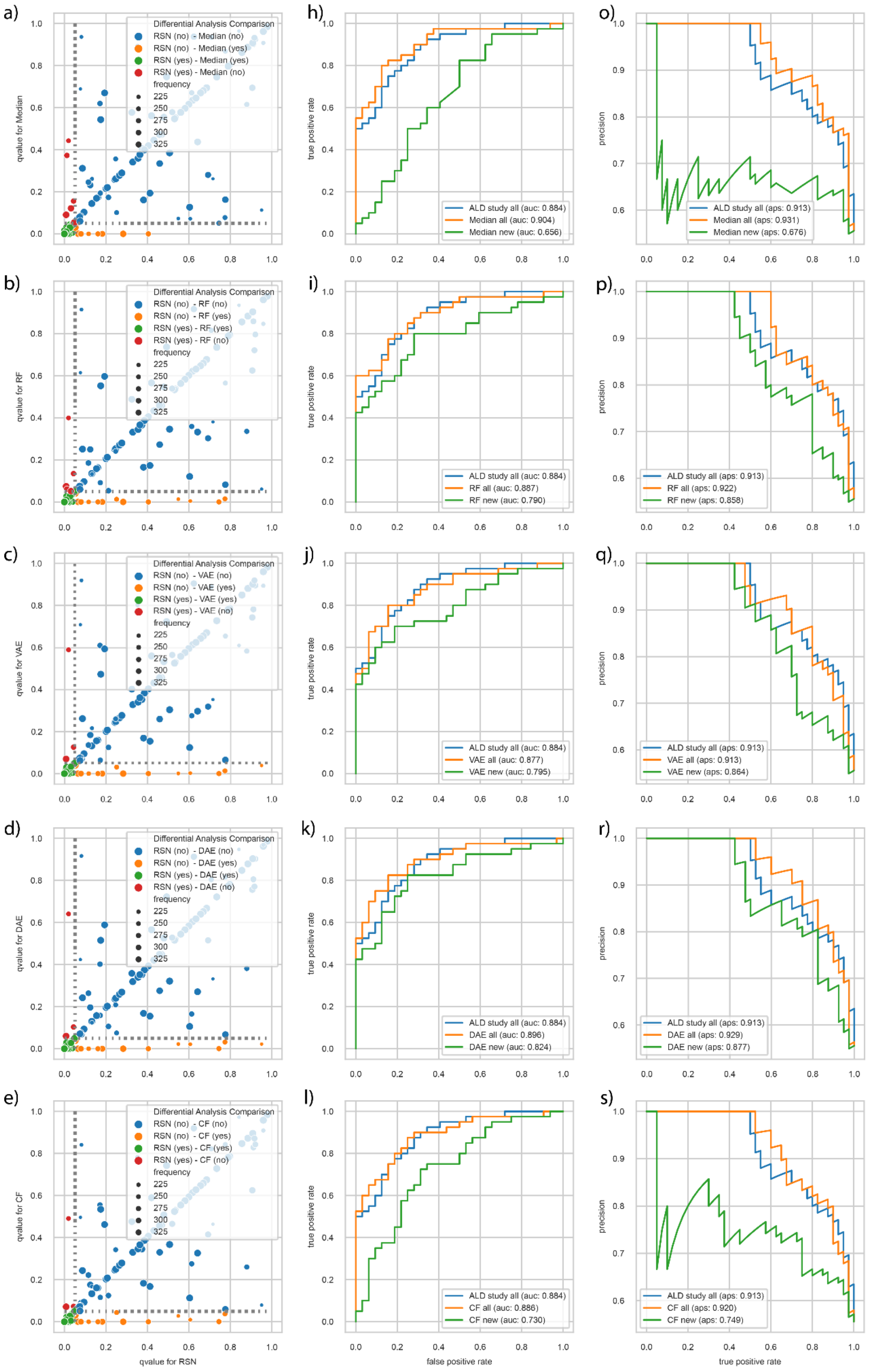
a)
Differential Analysis Comparison
RSN (no) - Median (no)
RSN (no) - Median (yes)
RSN (yes) - Median (yes)
RSN (yes) - Median (no)
frequency
225
250
275
300
325
qvalue for Median
b)
Differential Analysis Comparison
RSN (no) - RF (no)
RSN (no) - RF (yes)
RSN (yes) - RF (yes)
RSN (yes) - RF (no)
frequency
225
250
275
300
325
qvalue for RF
c)
Differential Analysis Comparison
RSN (no) - VAE (no)
RSN (no) - VAE (yes)
RSN (yes) - VAE (yes)
RSN (yes) - VAE (no)
frequency
225
250
275
300
325
qvalue for VAE
d)
Differential Analysis Comparison
RSN (no) - DAE (no)
RSN (no) - DAE (yes)
RSN (yes) - DAE (yes)
RSN (yes) - DAE (no)
frequency
225
250
275
300
325
qvalue for DAE
e)
Differential Analysis Comparison
RSN (no) - CF (no)
RSN (no) - CF (yes)
RSN (yes) - CF (yes)
RSN (yes) - CF (no)
frequency
225
250
275
300
325
qvalue for CF
qvalue for RSN
h)
true positive rate
ALD study all (auc: 0.884)
Median all (auc: 0.904)
Median new (auc: 0.656)
i)
ALD study all (auc: 0.884)
RF all (auc: 0.887)
RF new (auc: 0.790)
j)
ALD study all (auc: 0.884)
VAE all (auc: 0.877)
VAE new (auc: 0.795)
k)
ALD study all (auc: 0.884)
DAE all (auc: 0.896)
DAE new (auc: 0.824)
l)
ALD study all (auc: 0.884)
CF all (auc: 0.886)
CF new (auc: 0.730)
false positive rate
o)
precision
ALD study all (aps: 0.913)
Median all (aps: 0.931)
Median new (aps: 0.676)
p)
ALD study all (aps: 0.913)
RF all (aps: 0.922)
RF new (aps: 0.858)
q)
ALD study all (aps: 0.913)
VAE all (aps: 0.913)
VAE new (aps: 0.864)
r)
ALD study all (aps: 0.913)
DAE all (aps: 0.929)
DAE new (aps: 0.877)
s)
ALD study all (aps: 0.913)
CF all (aps: 0.920)
CF new (aps: 0.749)
true positive rate

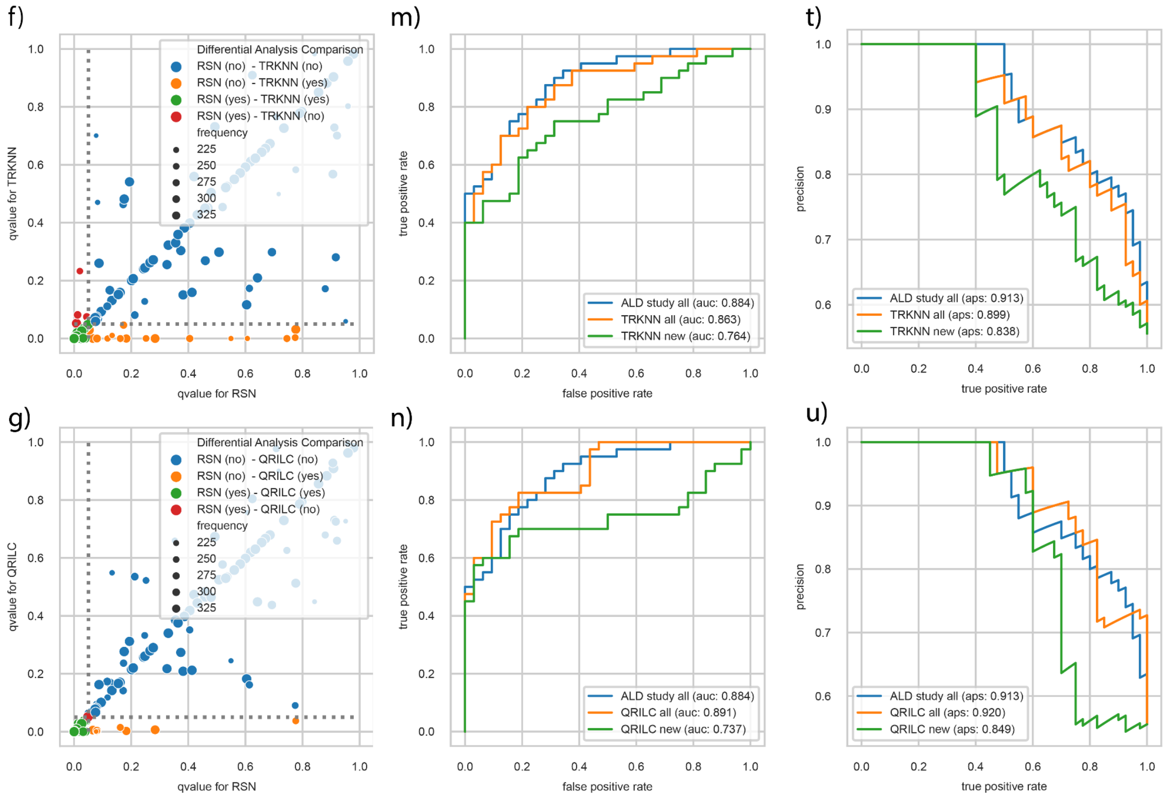

Supplementary Figure 8. Differential analysis comparisons and logistic regression performance using models. a-g) Comparing qvalues based on RSN imputation (x-axis) to the ones based on median, RF, DAE, VAE, CF, TRKNN and QRILC imputations (y-axis) for the 313 protein groups included in Niu. et al 2021. **h-n)** AUROC of logistic regression on test data allowing selection of original ALD study included 313 protein groups and RSN imputation (blue) to all 377 (orange) or only the 64 new protein groups included here (green). **o-u)** As for h-n showing PRAUC curves for the logistic regression on test data.

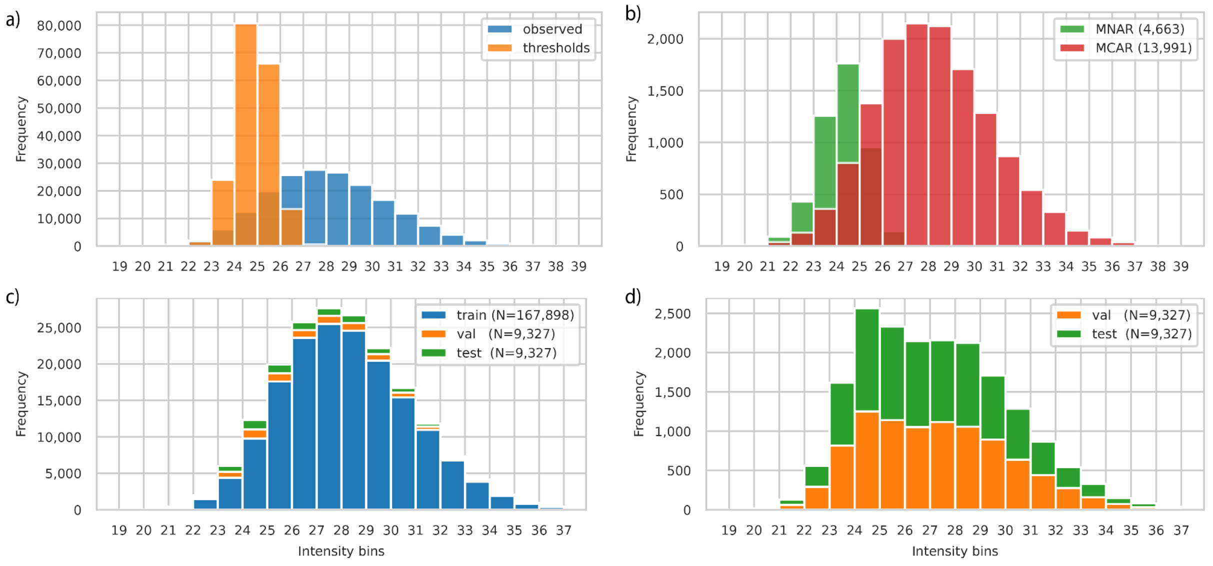

Supplementary Figure 9. Stacked histogram of train, validation and test split for protein groups of smaller development dataset with a share of 25 percent MNAR in the simulated missing values. a) Histogram of thresholds (orange) for each observed (blue) value for MNAR selection procedure. **b)** Sampled MNAR and MCAR simulated missing values. **c)** Stack histogram of all three splits. **d)** Validation and test data split stacked. Bins were created per integer of logarithm two transformed intensities. Number of intensities per split in parentheses.

Supplementary Tables

Method	Package	Source	Description
CF	pimms-learn	PyPI	Collaborative Filtering
DAE	pimms-learn	PyPI	Denoising Autoencoder
VAE	pimms-learn	PyPI	Variational Autoencoder
KNN	Scikit-learn	PyPI	K nearest neighbor imputation
RSN	pimms-learn	PyPi	Downshifted normal distribution (per sample), as PI
Median	pimms-learn	PyPI	Imputation by feature median
ZERO	-	-	replace NA with 0
MINIMUM	-	-	replace NA with global minimum
COLMEDIAN	e1071	CRAN	replace NA with column median
ROWMEDIAN	e1071	CRAN	replace NA with row median
KNN_IMPUTE	impute	BIOCONDUCTOR	k nearest neighbor imputation
SEQKNN	SeqKnn	tar file	Sequential k- nearest neighbor imputation starts with feature with least missing values and re-use imputed values for not yet imputed features
BPCA	pcaMethods	BIOCONDUCTOR	Bayesian PCA missing value imputation
SVDMETHOD	pcaMethods	BIOCONDUCTOR	replace NA initially with zero, use k most significant eigenvalues using Singular Value Decomposition for imputation until convergence
LLS	pcaMethods	BIOCONDUCTOR	Local least squares imputation of a feature based on k most correlated features
MLE	norm	CRAN	Maximum likelihood estimation
QRILC	imputeLCMD	CRAN	quantile regression imputation of left-censored data, i.e. by random draws from a truncated distribution which parameters were estimated by quantile regression
MINDET	imputeLCMD	CRAN	replace NA with q-quantile minimum in a sample
MINPROB	imputeLCMD	CRAN	replace NA by random draws from q-quantile minimum centered distribution
IRM	VIM	CRAN	iterativ robust model-based imputation (one feature at at time)
IMPSEQ	rrcovNA	CRAN	Sequential imputation of missing values by minimizing the determinant of the covariance matrix with imputed values
IMPSEQROB	rrcovNA	CRAN	Sequential imputation of missing values using robust estimators
MICE-NORM	mice	CRAN	Multivariate Imputation by Chained Equations (MICE) using Bayesian linear regression
MICE-CART	mice	CRAN	Multivariate Imputation by Chained Equations (MICE) using regression trees
TRKNN	-	script	truncation k-nearest neighbor imputation
RF	missForest	CRAN	Random Forest imputation (one feature at a time)
PI	-	-	Downshifted normal distribution (per sample)
GSIMP	-	script	QRILC initialization and iterative Gibbs sampling with generalized linear models (glmnet)
MSIMPUTE	msImpute	BIOCONDUCTOR	Missing at random algorithm using low rank approximation
MSIMPUTE MNAR	msImpute	BIOCONDUCTOR	Missing not at random algorithm using low rank approximation

Supplementary Table 1. **Methods overview in current comparison**. Sources for packages are mainly PyPI for PIMMS, CRAN and Bioconductor for R packages. Some R packages require custom installations. Find the up-to-date overview on GitHub.

Gene	Freq. observed	CF	DAE	Median	None	QRILC	RF	TRKNN	VAE
S100A9	168	10	10	0	0	0	10	10	10
PEBP4	124	10	10	0	0	0	10	10	10
GLIPR2	167	10	10	0	0	3	10	10	10
IGKV1-37	156	10	10	0	0	9	10	10	10
HSPG2	137	10	1	10	0	10	7	10	5
PRDX2	110	10	10	0	0	4	9	10	10
KRT25	148	3	9	0	0	0	0	10	7
RPS6KA3	179	0	0	0	0	4	0	0	0
TLN1	106	0	3	0	0	0	1	0	9
CCT3	108	0	2	0	0	0	0	0	2
ALDOA	102	10	10	0	0	0	8	10	10
ANK1	112	10	10	0	0	8	10	10	10
RAB2B	187	10	10	0	0	0	10	10	10
PZP	200	10	4	0	0	0	7	10	10
HSPA1B;HSPA1A	107	10	10	0	0	0	10	10	10
AMY1C	92	10	10	10	0	7	10	10	9
ALCAM	124	10	10	0	0	10	10	10	10
CCT6A	139	0	2	0	0	0	0	0	0
GSTO1	111	0	1	0	0	3	0	10	0
C16orf46	195	0	1	0	0	0	0	0	0
CA3	152	0	8	0	0	0	1	10	7
P4HB	111	0	3	0	0	0	3	10	2
REG1A;REG1B	107	7	0	0	0	1	6	10	3
FLNA	99	10	9	0	0	0	9	10	9
ZNF511-PRAP1;PRAP1	148	0	5	0	0	0	4	10	6
SOD3	117	0	0	0	0	0	0	10	0
TMSB4X	125	0	0	0	0	0	0	10	0
DPP4	110	0	0	0	0	0	0	10	1
ICAM2	132	10	10	0	0	10	10	10	9
KRT10	117	0	1	0	0	0	0	10	1
DCD	94	0	0	0	0	0	0	10	0
RARRES2	110	0	2	0	0	0	1	10	6
ITSN1	124	10	8	0	0	2	1	0	4
SAA2	130	10	8	0	0	0	10	0	5
APMAP	196	0	0	0	0	2	0	10	0
NPEPPS	180	0	0	0	0	1	0	0	0
PPIA	185	0	0	0	0	2	0	0	0
FAH	115	0	1	0	0	0	0	0	1

Supplementary Table 2. Counts of how often 54 differentially abundant protein groups which were newly included were found by any included model to be significant running the analysis 10 times. No imputation (None), Median and TRKNN imputation did not change between runs (**Supplementary Data 12**).
