# Supplementary material for: Imputation of label-free quantitative mass spectrometry-based proteomics data using self-supervised deep learning
Source: Nat Commun. 2024 Jun 26;15:5405. doi: 10.1038/s41467-024-48711-5 (PMC11208500; doi:10.1038/s41467-024-48711-5)
Supplement: Supplementary file 2 — Reporting Summary [file 41467_2024_48711_MOESM2_ESM.pdf]

nature portfolio

Corresponding author(s): Simon Rasmussen

Last updated by author(s): Apr 22, 2024

Reporting Summary

Nature Portfolio wishes to improve the reproducibility of the work that we publish. This form provides structure for consistency and transparency in reporting. For further information on Nature Portfolio policies, see our Editorial Policies and the Editorial Policy Checklist.

Statistics

For all statistical analyses, confirm that the following items are present in the figure legend, table legend, main text, or Methods section.

n/a	Confirmed	
☐	☒	The exact sample size (*n*) for each experimental group/condition, given as a discrete number and unit of measurement
☐	☒	A statement on whether measurements were taken from distinct samples or whether the same sample was measured repeatedly
☐	☒	The statistical test(s) used AND whether they are one- or two-sided<br>*Only common tests should be described solely by name; describe more complex techniques in the Methods section.*
☐	☒	A description of all covariates tested
☐	☒	A description of any assumptions or corrections, such as tests of normality and adjustment for multiple comparisons
☐	☒	A full description of the statistical parameters including central tendency (e.g. means) or other basic estimates (e.g. regression coefficient) AND variation (e.g. standard deviation) or associated estimates of uncertainty (e.g. confidence intervals)
☐	☒	For null hypothesis testing, the test statistic (e.g. *F*, *t*, *r*) with confidence intervals, effect sizes, degrees of freedom and *P* value noted<br>*Give P values as exact values whenever suitable.*
☒	☐	For Bayesian analysis, information on the choice of priors and Markov chain Monte Carlo settings
☒	☐	For hierarchical and complex designs, identification of the appropriate level for tests and full reporting of outcomes
☐	☒	Estimates of effect sizes (e.g. Cohen's *d*, Pearson's *r*), indicating how they were calculated

Our web collection on statistics for biologists contains articles on many of the points above.

Software and code

Policy information about availability of computer code

Data collection	MaxQuant v1.6.12, Spectronaut v15.4
Data analysis	Continous integration using specified environment file and testing: https://github.com/RasmussenLab/pimms/blob/HEAD/environment.yml<br><br>Otherwise versions of software for submission are:<br>- python=3.8.13<br>- numpy=1.20.1<br>- pandas=1.4.3<br>- scipy=1.7.3<br>- matplotlib=3.3.4<br>- plotly=5.9.0<br>- seaborn=0.12.2<br>- pytorch>=1.13<br>- scikit-learn=1.0.2<br>- fastai=2.5.3<br>- pytorch-cuda=11.7<br>- umap-learn=0.5.3<br>- pingouin=0.5.2<br>- statsmodels=0.12.2

- snakemake-minimal=7.12.0
- jupyterlab=3.3.2
- ipython=7.22.0
- pytest=7.1.2
- jupytext=1.14.0
- mrmr_selection=0.2.5

Some of these packages have OS specific dependencies which are not listed explicitly.

For the R packages we used R 4.1 or higher (depends on operating system):
- e1701=1.7.7
- impute=1.60.0
- pcaMethods=1.78.0
- SeqKNN=1.0.1
- norm=1.0-9.5
- imputeLCMD=2.1
- VIM=6.1.0
- rrcovNA=0.5
- mice=3.13.0
- missForest=1.4
- msImpute=1.12.0

For manuscripts utilizing custom algorithms or software that are central to the research but not yet described in published literature, software must be made available to editors and reviewers. We strongly encourage code deposition in a community repository (e.g. GitHub). See the Nature Portfolio guidelines for submitting code & software for further information.

Data

Policy information about availability of data

All manuscripts must include a data availability statement. This statement should provide the following information, where applicable:

- Accession codes, unique identifiers, or web links for publicly available datasets
- A description of any restrictions on data availability
- For clinical datasets or third party data, please ensure that the statement adheres to our policy

The mass spectrometry proteomics data have been deposited to the ProteomeXchange Consortium via the PRIDE68 partner repository with the dataset identifier PXD042233. A manuscript describing these data has been submitted to Scientific Data and is available as a pre-print at https://doi.org/10.21203/rs.3.rs-3083547/v2.

Additionally, the HeLa data is uploaded to the ERDA file sharing service:

development datasets: https://erda.ku.dk/archives/393b4ee7e16176f2a668d96d5f636ce3/published-archive.html
example dataset: https://github.com/RasmussenLab/pimms/tree/dev/project/data/dev_datasets/HeLa_6070 (used per default for workflow)

The clinical data is not freely available, but can be requested as specified by Niu et. al.2 : “The full proteomics datasets and histologic scoring generated and/or analyzed (...) are available (...) upon request, to Odense Patient Data Exploratory Network (open@rsyd.dk) with reference to project ID OP_040. Permission to access and analyze data can be obtained following approval from the Danish Data Protection Agency and the ethics committee for the Region of Southern Denmark.”

Research involving human participants, their data, or biological material

Policy information about studies with human participants or human data. See also policy information about sex, gender (identity/presentation), and sexual orientation and race, ethnicity and racism.

Reporting on sex and gender	Information on sex was self-reported and checked using X-chromosome data using the Plink software. Boys and girls were well-balanced in the study (55% girls and 45% boys). Sex-based analysis was performed in testing the effects of sex on the plasma proteome. If the term gender is used, it can be seen synonyms to sex.
Reporting on race, ethnicity, or other socially relevant groupings	No specific groupings were introduced.
Population characteristics	The human participant data is described as before in Niu. et. al. 2022. See full reporting summary under https://static-content.springer.com/esm/art%3A10.1038%2Fs41591-022-01850-y/MediaObjects/41591_2022_1850_MOESM2_ESM.pdf. We repeat the statements for the GALA-ALD cohort (n=459) which was used for our analysis:<br><br>GALA-ALD patient characteristics (n=459): Median age 57 (IQR 13) years, 76% male, BMI 27.4 (IQR 6.7) kg/m2, MELD-score 6 (IQR 2, range 6-18), liver stiffness measured by FibroScan 6.5 (IQR 6.8, range 1.5-75) kPa. Liver fibrosis stage from biopsies: F0/1/2/3/4 = 36/124/106/27/67. Not all biopsies contained enough tissue for adequate assessment of steatosis, ballooning and lobular inflammation, the following does therefore encompass 352 patients.94 patients have severe fibrosis or cirrhosis (F3 n=27, 29%; F4 n=67, 71%).

Recruitment	For the GALA-ALD cohort (n=459), we recruited patients consecutively through standard referrals to three outpatient liver clinics, from two municipal alcohol rehabilitation centers, and through a community call to screen for alcohol-related liver disease in the Region of Southern Denmark. Inclusion criteria in the GALA-ALD cohort are a history of excessive use of alcohol for more than 1 year (>24 g per day for women and >36 g per day for men), age 18–75 years, and informed consent to undergo a liver biopsy. We excluded patients with evidence of decompensated cirrhosis (obvious ascites, known esophageal varices, prior decompensation), concurrent liver disease other than alcohol-related, severe alcoholic hepatitis, debilitating disease with an expected survival of less than one year, hepatic congestion or cholestasis evidenced by ultrasound, or inability to comply with the study protocol.
Ethics oversight	The study protocol was approved by ethics committee for the Region of Southern Denmark (ethical IDs: S-20160006G, S-20120071, S-20160021, S-20170087) and registered with the Danish Data Protection Agency (13/8204, 16/3492, 18/22692).

Note that full information on the approval of the study protocol must also be provided in the manuscript.

Field-specific reporting

Please select the one below that is the best fit for your research. If you are not sure, read the appropriate sections before making your selection.

☒ Life sciences ☐ Behavioural & social sciences ☐ Ecological, evolutionary & environmental sciences

For a reference copy of the document with all sections, see nature.com/documents/nr-reporting-summary-flat.pdf

Life sciences study design

All studies must disclose on these points even when the disclosure is negative.

Sample size	The experimental data from the Alcoholic Liver Disease (ALD) is described as before in Niu. et. al. 2022. See reporting summary under https://static-content.springer.com/esm/art%3A10.1038%2Fs41591-022-01850-y/MediaObjects/41591_2022_1850_MOESM2_ESM.pdf:<br><br>When designing the study protocol (prior to recruiting participants), we planned to work with multiomics datasets using systems biology. Consequently, the sample size was based on experience from prior -omics studies in humans. To detect small effect sizes under parametric assumptions at 0.05 significance level and 0.75 statistical power, we estimate that 364 samples will be required for comparing proteomics profiles using statistical power analysis principles outlined by Cohen (Statistical power analysis for the behavioral sciences, 2nd edition, 1988). To adjust for non-parametric tests, the corresponding minimum required samples size using Pitman's Asymptotic Relative Efficiency is 381 participants. To provide us with enough statistical power to detect small effect sizes even when testing a handful of outcome variables and controlling the false discovery rate at 0.05, while striking a balance between exclusions/drop-outs and not including an unnecessary amount of participants, we planned to enroll more than 400 alcohol over-users and less than 500. However, we would like to emphasize that traditional ways of calculating statistical power do not strictly apply to omics research, where strong patterns have been identified using small sample sizes.
Data exclusions	Data exclusion is a of the overall comparison framework. We excluded features which were present in less than 25 percent of the samples entirely. In general, the cutoffs for sample and feature completeness vary depending on the design of the comparisons.<br><br>Only individual with a complete set of covariates and an histological kleiner disease score were taken into consideration for differential analysis. This let to the exclusion of 10 individuals with missing data as was done in the original study for differential analysis. Otherwise all available data was used.
Replication	In the original study, the identified protein marker panels and the derived logistic regression models for predicting liver fibrosis, inflammation and steatosis in the discovery cohort were validated in an independent cohort with N=63.
Randomization	The proteomics data acquisition of samples was randomized to avoid bias. Randomization was performed using the RAND formula in Excel.
Blinding	The pathologist who scored the liver biopsy samples were blinded to clinical patient parameters other than age and gender.

Reporting for specific materials, systems and methods

We require information from authors about some types of materials, experimental systems and methods used in many studies. Here, indicate whether each material, system or method listed is relevant to your study. If you are not sure if a list item applies to your research, read the appropriate section before selecting a response.

Materials & experimental systems

n/a	Involved in the study
☒	☐ Antibodies
☐	☒ Eukaryotic cell lines
☒	☐ Palaeontology and archaeology
☒	☐ Animals and other organisms
☐	☒ Clinical data
☒	☐ Dual use research of concern
☒	☐ Plants

Methods

n/a	Involved in the study
☒	☐ ChIP-seq
☒	☐ Flow cytometry
☒	☐ MRI-based neuroimaging

Eukaryotic cell lines

Policy information about cell lines and Sex and Gender in Research

Cell line source(s)	The quality control runs were performed with the in house HeLa cell line. Protocols differ slightly by group and researcher for the runs in our collection. A detailed disaggregation of each run is not possible. The data is only used to develop the models and not to create biological insight. Differences in measurements of a very similar lysate are envisioned to provide a good noise for evaluating the learning setups.<br><br>User of the software will not use data used for establishing the setup, but will rerun it on their own data.
Authentication	Not a controlled cellline, but produced in-house for quality control of mass spectromer.
Mycoplasma contamination	To avoid perpetual contamination the quality control HeLa cellline is tested once a month for mycoplasma contamination.
Commonly misidentified lines (See ICLAC register)	Only HeLa was used.

Clinical data

Policy information about clinical studies

All manuscripts should comply with the ICMJEguidelines for publication of clinical research and a completedCONSORT checklist must be included with all submissions.

Clinical trial registration	We did not do the clinical trial ourselves, but reused the data from Niu. et. al 2022. See reporting summary under https://static-content.springer.com/esm/art%3A10.1038%2Fs41591-022-01850-y/MediaObjects/41591_2022_1850_MOESM2_ESM.pdf<br><br>The study was registered with Odense Patient Data Exploratory Network under study identification number OP_040 (open.rsyd.dk/OpenProjects/da/openProjectList.jsp). The study protocol for the ALD validation cohort was registered at clinicaltrials.gov ID NCT03308916.
Study protocol	Study protocol can be obtained by contact to open@rsyd.dk
Data collection	GALA-ALD: Single center data collection, at Center for Liver Research, Odense University Hospital, Denmark. Inclusion and recruitment period: April 2013 to September 2018.
Outcomes	Primary outcome was correlation between the plasma proteome and liver fibrosis assessed by the diagnostic gold standard liver biopsy scored according to Kleiner fibrosis stage.

Plants

Seed stocks	*Report on the source of all seed stocks or other plant material used. If applicable, state the seed stock centre and catalogue number. If plant specimens were collected from the field, describe the collection location, date and sampling procedures.*
Novel plant genotypes	*Describe the methods by which all novel plant genotypes were produced. This includes those generated by transgenic approaches, gene editing, chemical/radiation-based mutagenesis and hybridization. For transgenic lines, describe the transformation method, the number of independent lines analyzed and the generation upon which experiments were performed. For gene-edited lines, describe the editor used, the endogenous sequence targeted for editing, the targeting guide RNA sequence (if applicable) and how the editor was applied.*
Authentication	*Describe any authentication procedures for each seed stock used or novel genotype generated. Describe any experiments used to assess the effect of a mutation and, where applicable, how potential secondary effects (e.g. second site T-DNA insertions, mosiacism, off-target gene editing) were examined.*
